# Heritability and genome-wide association of swine gut microbiome features with growth and fatness parameters

**DOI:** 10.1038/s41598-020-66791-3

**Published:** 2020-06-23

**Authors:** Matteo Bergamaschi, Christian Maltecca, Constantino Schillebeeckx, Nathan P. McNulty, Clint Schwab, Caleb Shull, Justin Fix, Francesco Tiezzi

**Affiliations:** 1 Department of Animal Science, North Carolina State University, Raleigh, NC 27695 USA; 2 Matatu, Inc., 4340 Duncan Ave., Suite 211, St. Louis, MO 63110 USA; 3 The Maschhoffs LLC, Carlyle, IL 62231 USA

**Keywords:** Agricultural genetics, Animal breeding, Genetic association study, Genetic markers, Genotype, Heritable quantitative trait, Microbial genetics, Quantitative trait

## Abstract

Despite recent efforts to characterize longitudinal variation in the swine gut microbiome, the extent to which a host’s genome impacts the composition of its gut microbiome is not yet well understood in pigs. The objectives of this study were: i) to identify pig gut microbiome features associated with growth and fatness, ii) to estimate the heritability of those features, and, iii) to conduct a genome-wide association study exploring the relationship between those features and single nucleotide polymorphisms (**SNP**) in the pig genome. A total of 1,028 pigs were characterized. Animals were genotyped with the Illumina PorcineSNP60 Beadchip. Microbiome samples from fecal swabs were obtained at weaning (**Wean**), at mid-test during the growth trial (**MidTest**), and at the end of the growth trial (**OffTest**). Average daily gain was calculated from birth to week 14 of the growth trial, from weaning to week 14, from week 14 to week 22, and from week 14 to harvest. Backfat and loin depth were also measured at weeks 14 and 22. Heritability estimates (±SE) of Operational Taxonomic Units ranged from 0.025 (±0.0002) to 0.139 (±0.003), from 0.029 (±0.003) to 0.289 (±0.004), and from 0.025 (±0.003) to 0.545 (±0.034) at Wean, MidTest, and OffTest, respectively. Several SNP were significantly associated with taxa at the three time points. These SNP were located in genomic regions containing a total of 68 genes. This study provides new evidence linking gut microbiome composition with growth and carcass traits in swine, while also identifying putative host genetic markers associated with significant differences in the abundance of several prevalent microbiome features.

## Introduction

Host-associated microbes influence health and disease profoundly^1^. The microorganisms residing in the gut live in close contact with each other and establish many symbiotic relationships with the host^2^. These gut communities are influenced by many environmental factors, such as lifestyle, age, sex, and diet^3,4^. Few studies in humans have investigated the effect of the host on the abundance of specific gut microbes^5,6^. In pigs, a small but growing body of literature has focused on investigating the host effect including genome and feed efficiency on the composition of the gut microbiome^7–9^. Researchers have previously explored how the pig gut microbiome is impacted by environmental factors such as nutrition^10^, weaning-associated stressors^11–13^, and management practices^14,15^. Also, pigs are an important model for studying the interaction between host genetics and gut microbiome in humans, given the high similarity of the digestive system between the two species. Compared to humans, domestic pigs can be reared under similar conditions and may be fed with controlled diets. Understanding the relationship between host genetics and gut microbiome in pigs might reveal new insights about how the gut microbiome of humans is related to complex traits^16–18^. Elucidating the host effect on the gut microbiome involves several steps, such as investigating the composition of the microbiome as well as identifying potential genetic variants underlying variation in the microbiome^19^. The research reported herein is trying to partially address some of these questions in swine. The study relies on a large sampling population with both genomic and microbiome information collected. Specifically in this paper we report: i) fecal microbiome features whose abundances are significantly associated with host growth and fatness parameters; ii) heritability estimates for such taxa; and, iii) SNP determined in a genome-wide association study to be significantly associated with differences in the relative abundance of such taxa.

## Results

The distribution of taxonomic abundances at various levels for the day after weaning (**Wean**), 15 weeks post-weaning (**MidTest**), and 22 weeks post-weaning (**OffTest**) has been previously reported in Lu *et al*.^19^ and Maltecca *et al*.^20^. Since the aim of this study was not to provide the ecological landscape of the population measured, in this study we reported the family level. The representation of bacterial families abundance is reported in Supplementary Fig. 1. Dominant families were *Clostridiaceae* and *Prevotellaceae* for all three census times. In this study we identified fecal microbiome operational taxonomic units (**OTUs**) associated with 8 traits: 4 measures of weight gain, as well as two backfat thickness, and loin depth measures obtained at weeks 14 and 22 of the trial. We estimated the heritability of OTUs measured in samples of rectal swab of crossbred pigs. Following, we performed a Genome-Wide Association Study between heritable OTUs and SNP.

### Identifying OTUs associated with pig performance

**Figure 1:**
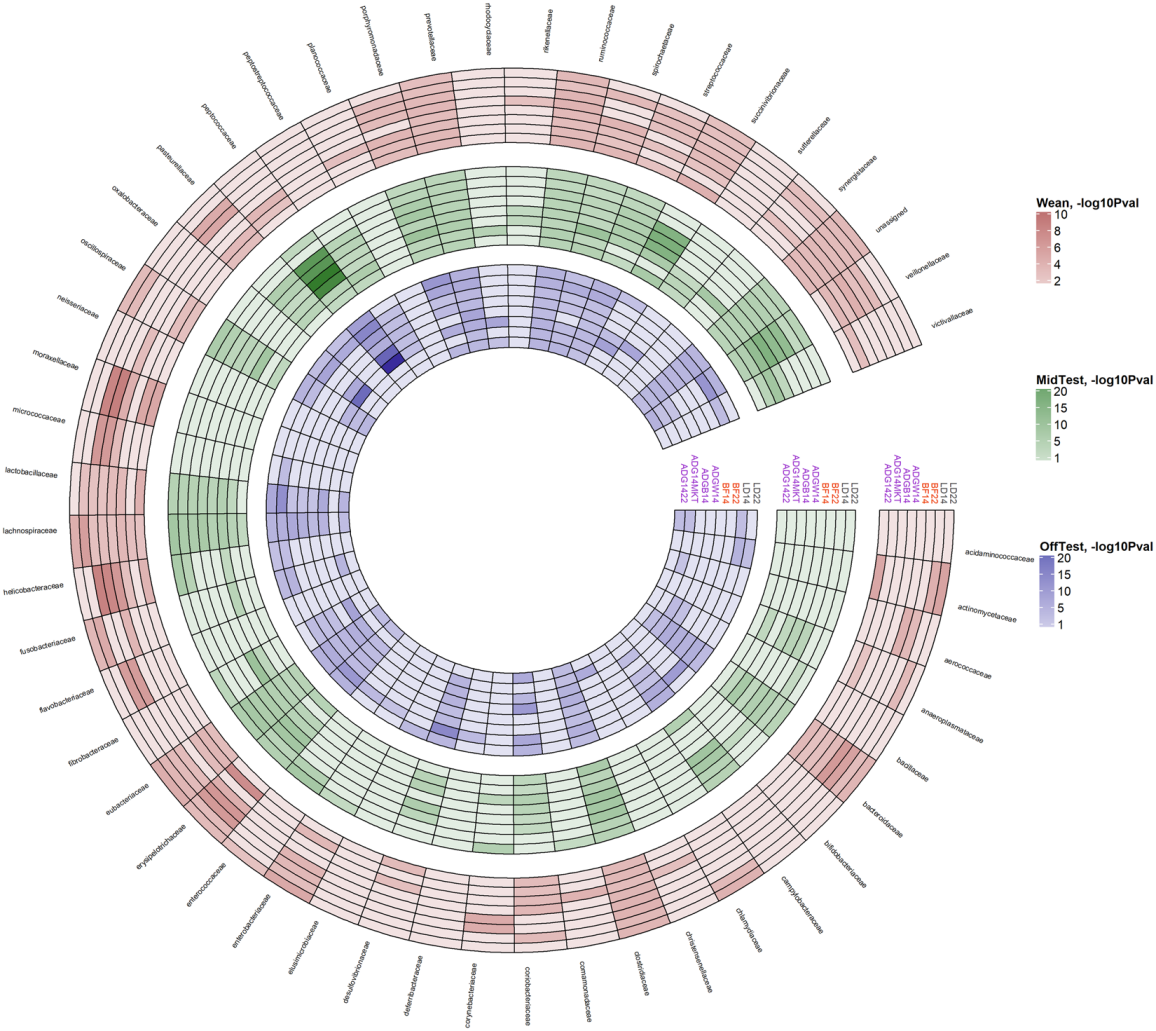
Association between growth and fatness with operational taxonomic unit at family level at Wean, MidTest, and OffTest. The color gradient indicates the level of significance -log_10_(*P*-value). ADG_B-14_ = average daily gain from birth to week 14 post-weaning; ADG_W-14_ = average daily gain from weaning to week 14 post-weaning; ADG_14–22_ = average daily gain from weeks 14 to 22 post-weaning; ADG_14-MKT_ = average daily gain from week 14 post-weaning to market (dark violet); BF = back fat thickness measured at 14 or 22 weeks post-weaning (orange); LD = loin depth measured at 14 or 22 weeks post-weaning (black).

We tested the association between OTU abundance and average daily gain calculated as difference in live weight from birth to week 14 (**ADG**_**B-14**_), from weaning to week 14 (**ADG**_**W-14**_), from week 14 to week 22 (**ADG**_**14–22**_) and from week 14 to harvest (**ADG**_**14-MKT**_), back fat thickness and loin depth recorded at weeks 14 and 22 post-weaning (**BF**_**14**_, **BF**_**22**_, **LD**_**14**_, **LD**_**22**_ respectively). Figure 1 depicts the *–log*_10_*(P-value)* of the association between OTUs at family level with growth and fatness when considering Wean, MidTest, and OffTest OTU abundances. *Moraxellaceae*, *Oxalobacteraceae*, and *Peptococcaceae* were highly associated with growth and fatness across time points (Fig. 1). Both positive and negative associations between those bacterial families and growth and fatness parameters were highly significant. Additional information can be found in Supplementary Table 1, where taxonomic classification, OTU effect on each trait, standard error, variance proportion, and level of significance of the associations between individual OTU and ADG_B-14_, ADG_W-14_, ADG_14–22_, ADG_14-MKT_, BF_14_, BF_22_, LD_14_, and LD_22_ at different time points are reported.

At an FDR of 5%, there were 334 OTUs significantly associated with growth and fatness parameters. The 9% of those did not have an assigned phylum. Of the rest, most were assigned to 4 prominent phyla (*Firmicutes* 64%; *Bacteroidetes* 16%; *Proteobacteria* 4%; and *Actinobacteria* 2%), while 5% were assigned to phyla with lower abundance. The 52.0% of the 334 OTUs could not be reliably assigned to a specific genus. Of the remainder, 7.0% were in *Clostridium*, 5.0% in *Prevotella*, 4.0% in *Lactobacillus*, 3.5% in *Eubacterium*, 3.0% in *Bacterioides*, 2.6% in *Ruminococcus*, 2.0% in *Streptococcus*, 1.5% in *Treponema*, 1.1% in *Coprococcus*, 1.0% in *Faecalibacterium*, and 17.3% in others 43 genera with lower abundance.

The OTUs significantly associated with growth and fatness parameters at Wean, MidTest, and OffTest numbered 47, 108, and 206, respectively. Of these, 2 (both *Firmicutes*) were common between Wean and MidTest. Nineteen were in common between MidTest and OffTest (11 *Firmicutes*, 5 *Proteobacteria*, 2 *Bacteroidetes*, and 1 unassigned). Four were in common between Wean and OffTest (2 *Proteobacteria*, 1 *Firmicutes*, and 1 unassigned).

Overall, 245, 115, and 26 OTUs were significantly associated with average daily gain, backfat, and loin depth at the three time points. Of the OTUs significantly associated with average daily gain traits, the 84.5% belonged to just 3 phyla: *Firmicutes* (66.2%); *Bacteroidetes* (13.4%); and *Proteobacteria* (4.9%). At the genus level, the 54.2% of them were unassigned, 5.0% were classified as *Prevotella*, 5% as *Clostridium*, 4.5% as *Lactobacillus*, 4.0% as *Eubacterium*, 3.9% as *Ruminococcus*, 2.4% as *Streptococcus*, and the 21% were classified into other 31 minor genera. One hundred and one of these 245 OTUs were positively associated with average daily gain, while the opposite trend was observed for the remaining OTUs.

Forty-six OTUs (27 *Firmicutes*, 8 *Bacteroidetes*, 5 *Proteobacteria*, 1 *Actinobacteria*, and 5 unassigned) were associated with ADG_B-14_ and ADG_W-14_, while 27 OTUs (18 *Firmicutes*, 4 *Bacteroidetes*, and 5 unassigned) were linked to both ADG_14–22_ and ADG_14-MKT_. Both positive and negative directions between those Phyla and average daily gain traits were found. The proportion of the total phenotypic variation explained by the OTUs associated with ADG_B-14_ and ADG_W-14_ ranged from 0.052 to 0.008 while ranged from 0.018 to 0.005 for ADG_14–22_ and ADG_14-MKT_ (Supplementary Table 1).

Of the 115 OTUs significantly associated with backfat traits, 89.7% belonged to 3 phyla *Firmicutes* (64.7%); *Bacteroidetes* (20.0%) and *Proteobacteria* (5.0%). At the genus level, the 41.8% were unassigned, 10.4% were classified as *Clostridium*, 7.8% as *Prevotella*, 6.1% as *Bacteroides*, 4.3% as *Lactobacillus*, 4.3% as *Eubacterium*, 3.5% as *Succinivibrio*, and 21.8% OTUs were classified in other 17 minor genera. Sixty-one of these 115 OTUs were negatively associated with backfat thickness, meaning their increasing representation in the fecal microbiota corresponded with a decrease in subcutaneous fat deposition. The proportion of phenotypic variance explained by these 61 OTUs (68.8% *Firmicutes*, 9.8% *Proteobacteria*, 8.2% *Bacteroidetes*, 3.3% *Fibrobacteres*, 1.6% *Spirochaetes*, and 8.3% unassigned) ranged from to 0.034 to 0.007 (Supplementary Table 1). Ten *Firmicutes*, 2 *Bacteroidetes*, 3 *Proteobacteria*, and 2 unassigned phyla were found to affect both BF_14_ and BF_22_.

Only 26 OTUs were significantly associated with loin depth. Eighteen of them were classified as *Firmicutes*. Twelve of these 26 OTUs were positively associated with loin depth and explained a proportion of phenotypic variance from to 0.014 to 0.008 (Supplementary Table 1).

Two OTU were found to affect both LD_14_ and LD_22_. These OTUs were classified as *Firmicutes* and *Proteobacteria*.

Of the significant OTUs, 10 *Firmicutes*, 3 *Bacteroidetes*, and 1 *Proteobacteria* were associated with both ADG_14-MKT_ and BF_22_ which explained a proportion of phenotypic variance from 0.017 to 0.005 and from 0.053 to 0.008 for ADG_14-MKT_ and BF_22_, respectively (Supplementary Table 1). Ten *Firmicutes* were associated with ADG_B-14_, ADG_W-14_, and BF_14_. The proportion of phenotypic variance explained by these OTUs (max-min) was 0.051–0.011, 0.052–0.011, and 0.075–0.011 for ADG_B-14_, ADG_W-14_, and BF_14,_ respectively (Supplementary Table 1). In addition, 5 *Firmicutes* were associated with ADG_14–22_, ADG_14-MKT_, and BF_22_; 2 *Proteobacteria* and 1 *Firmicutes* were associated with growth (ADG_B-14_; ADG_W-14_) and fatness (BF_14_; BF_22_).

### Estimating the heritability of operational taxonomic units

**Figure 2:**
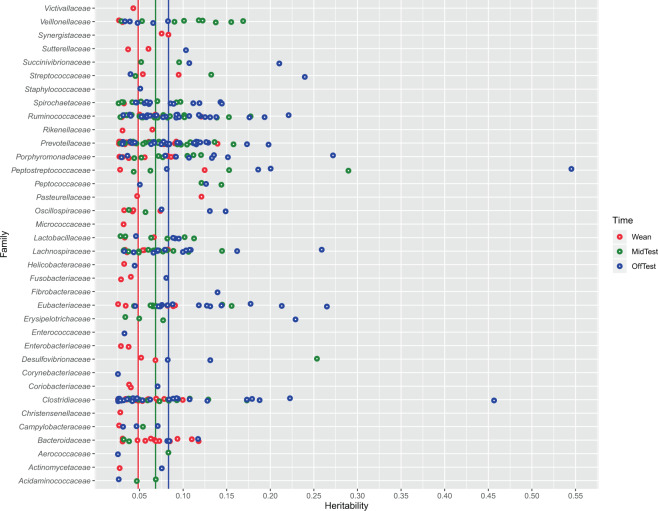
Heritability estimates for OTU abundances at Wean, MidTest, and OffTest. The vertical lines indicates the median value of heritability.

Variance components and heritability were estimated for 1,678 OTUs at Wean, MidTest, and OffTest. We identified 170, 261, and 366 heritable OTUs at Wean, MidTest, and OffTest, respectively. Results are presented in Fig. 2 and Supplementary Table 2. Operational taxonomic units heritability estimates (±SE) ranged from 0.025 (±0.0002) to 0.139 (±0.003), from 0.029 (±0.003) to 0.289 (±0.004), and from 0.025 (±0.003) to 0.545 (±0.034), for the three census points, respectively. The highest heritability estimates were 0.545 (±0.034) and 0.457 (±0.001) for OTU classified as *Peptostreptococcaceae* and *Clostridiaceae* at OffTest (Fig. 2).

Of the heritable OTUs, 43 were in common between Wean and MidTest. Of these, *Firmicutes* (24) and *Bacteroidetes* (15) were the most represented. Their heritability estimates ranged from 0.025 (±0.0005) to 0.289 (±0.004). One hundred sixty-six OTUs were heritable both at MidTest and OffTest. Of these, the majority belonged again to *Firmicutes* (110) and *Bacteroidetes* (40). Their heritability ranged from 0.025 (±0.0002) to 0.545 (±0.034). Thirty-two OTUs were heritable at all three census points. Of these, 19 were *Firmicutes*, 10 *Bacteroidetes*, 1 *Spirochaetes*, 1 *Proteobacteria*, and 1 unassigned.

Of the OTUs that were found to be significantly associated with growth and fatness, 52, 88, and 123 were heritable at Wean, MidTest, and OffTest, respectively. Of these OTUs, 15 were heritable both at Wean and MidTest, 60 were heritable at both MidTest and OffTest, and 12 were heritable at all three time points. Most of these OTUs belonged to the *Firmicutes* (9)*, Bacteroidetes* (1)*, Spirochaetes* (1), and *Proteobacteria* (1) phyla.

### Marker contribution to variation in operational taxonomic units

A genome-wide association study was conducted for the alpha diversity measures obtained at the three periods of the trial. Alpha diversity heritability of the gut microbiome for the three time points measured in this study was previously reported by Lu *et al*.^19^. Briefly, the alpha diversity had a low heritability at Wean (0.04 ± 0.04) and MidTest (0.15 ± 0.06) and moderate heritability at OffTest (0.33 ± 0.10)^19^. In this study, 7 markers were significantly associated with alpha diversity at Wean, 1 marker was significantly associated with alpha diversity at MidTest, and no significant associations were found between SNPs and alpha diversity at OffTest (Supplementary Fig. 2a–c).

**Figure 3:**
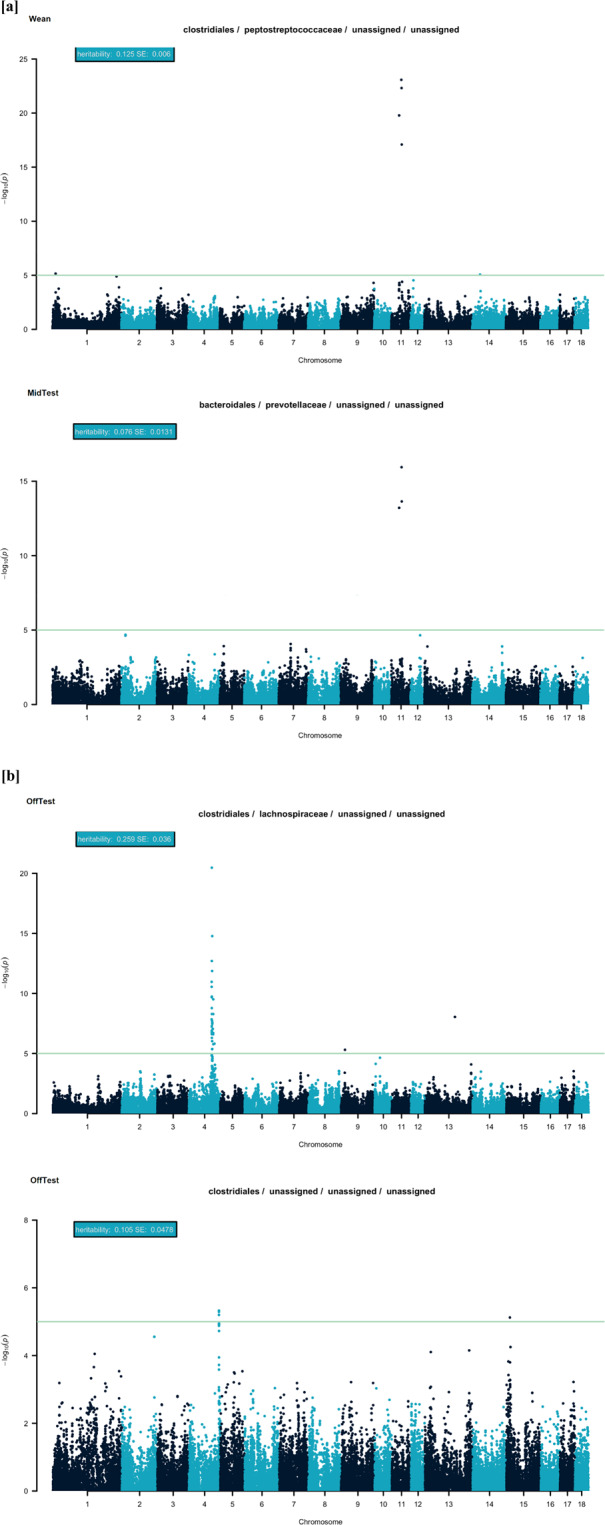
Manhattan plots for operational taxonomic units with at least one significant marker within Wean and MidTest [**a**], and OffTest [**b**]. The horizontal lines indicate the (*P* < 1 × 10^−5^) threshold for genome-wide significance.

There were 93 OTUs with at least one significant SNP after FDR correction across time points. They scattered across the 18 *Sus scrofa* chromosome (**SSC**). Regions with the strongest signal were on SSC4 and SSC11. Taxa exhibiting the greatest signals are reported in Fig. 3a,b, and all association results are reported in Supplementary Tables 3 and 4.

Seven markers spanning a region located from 34.7 to 47.0 Mb on SSC11 were significantly associated with several families at Wean, namely: *Prevotellaceae, Peptostreptococcaceae, Bacteroidaceae*, and *Clostridiaceae* (Fig. 3a and Supplementary Table 3). Similarly, 8 markers covering a region from 20.7 to 74.5 Mb on SSC11, as well as 9 markers in a region from 44.7 to 85.9 Mb, and 6 six markers in a region spanning from 107.1 to 148.3 Mb on SSC2 were significantly associated with OTUs at MidTest (Fig. 3a and Supplementary Table 3). These significant markers were associated with OTUs classified as *Lachnospiraceae*, *Prevotellaceae*, *Ruminococcaceae*, *Spirochaetaceae*, and *Succinivibrionaceae*. Of the significant genomic regions associated with OTUs at OffTest, one region located from 93.4 to 137.1 Mb on SSC4 and contained 44 markers significantly associated with OTU classified as *Eubacteriaceae* and *Lachnospiraceae* (Fig. 3b and Supplementary Table 3).

**Figure 4:**
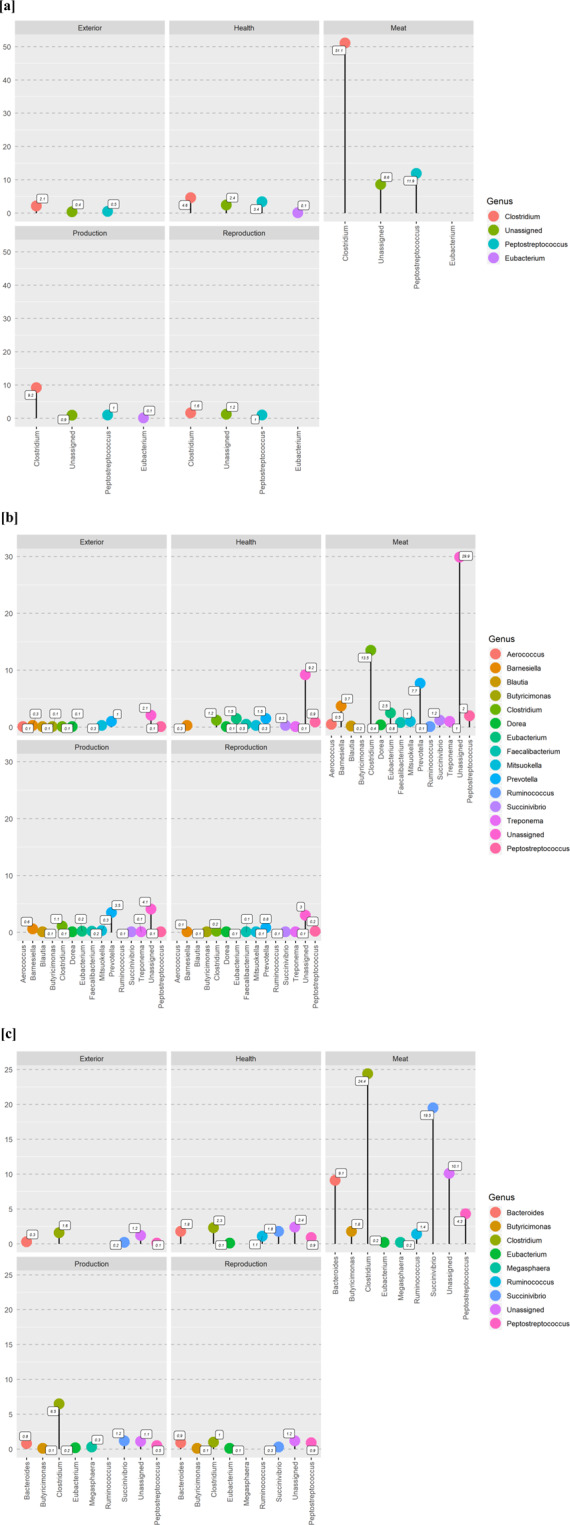
Quantitative trait loci (QTLs) identified for genome regions closed to significant markers (FDR 5%) associated with operational taxonomic units at three-time points: Wean [**a**], MidTest [**b**], and OffTest [**c**]. Y-axis of each plots indicate the % QTLdb hit.

Quantitative trait locus (**QTLs**) analysis revealed a link between OTU and specific regions of chromosomes. The genomic regions associated with alpha diversity and several OTUs co-localized with previously identified QTLs (Fig. 4a–c and Supplementary Table 5 and Supplementary Fig. 2a–c). We classified QTLs previously mapped in five major groups and expressed the co-localization as percentage of QTL hits with the markers identified in this study. Categories were: *“exterior”, “health”, “meat”, “production” and “reproduction”*. The highest number of QTL co-localizations occurred for *Clostridium* and *Prevotella* within the “meat” QTL group, followed by the QTL association for genera belonging to family *Peptostreptococcus* and *Succinivibrio* within the “meat” QTL group (Fig. 4a–c).

There were 68 reported genes in the Database for Annotation, Visualization and Integrated Discovery in close proximity (marker + or – 1 Mb) to the significant genomic regions and markers identified in this study. We compiled annotations for these genes (Supplementary Table 6). Among these genes were proteasome subunit alpha 1 (*PSMA1*, 44.64~44.75 Mb) located on SSC2 mainly enriched for proteasome and protein phosphatase 2, regulatory subunit B, beta (*PPP2R2B*, 147.9~148.8 Mb) located on SSC2 enriched for mRNA surveillance pathway, and sphingolipid signaling pathway (Supplementary Table 7). The gene encoding VANGL1 planar cell polarity protein 1 (*VANGL1*, 104.9~105.0 Mb) located on SSC4 was associated with the Wnt signaling pathway. The gene encoding DIS3 homolog, exosome endoribonuclease and 3'-5' exoribonuclease (*DIS3*, 45.04~45.08 Mb) located on SSC11 was associated with for RNA degradation (Supplementary Table 7).

## Discussion

In this study we found that gut microbiome diversity and specific constituents of the fecal microbiome were significantly associated with growth and fatness parameters in crossbred swine. The higher number of the associations was obtained for average daily gain and fatness. Three-hundred-thirty-four OTUs were significantly associated with growth and fatness parameters, many of which were classified as *Clostridium*, *Prevotella*, *Lactobacillus*, and *Eubacterium*. Niu *et al*.^21^ observed that the abundance of *Clostridium* within the pig gut microbiome changes in response to diets with different fiber fractions. Moreover, high abundance of *Clostridium* has been reported to be associated with increased fatness in pigs^22^ and the progression of obesity in mice^23,24^. However, species of the *Clostridium* have also been found not to be significantly associated with visceral fat mass in humans^25^ and with improved feed efficiency in poultry^26^. In our study 9 OTUs classified as *Clostridium* were associated with both backfat and loin depth at both MidTest and OffTest. Of these OTUs, six were negatively related and three positively related with fatness parameters and directionality consistent across time points. These results confirm the hypothesis of Maltecca *et al*.^20^ which found a higher amount of information in the gut microbiome of animals sampled in the later life stages to predict fatness parameters. Some OTUs classified as *Clostridium* showed a higher abundance in the gastrointestinal tract of pigs characterized by a higher backfat thickness compare to pigs with lower fat deposition capacity^27^. In our study, *Lactobacillus reuteri* was significantly associated with average daily gain and backfat. The genus *Lactobacillus* is often considered a beneficial group of bacteria in the mammalian gastrointestinal tract. *L. reuteri*, for example, competes against pathogens in the gut, improves immune function and antioxidant status, and alleviates weaning stress in piglets^28–30^. Furthermore, *L. reuteri* and *Lactobacillus amylovorus* exhibit probiotic properties that have been proven to promote growth^31–33^ and improve feed efficiency in farm animals^34,35^.

In this study, we also found a significant association between *Succinivibrio* and backfat. *Succinivibrio* species have been studied mostly in ruminants and to a lesser extent in monogastric animals. They reportedly play an important role in producing acetate and succinate^36^, which are essential for propionate synthesis as part of hepatic gluconeogenesis, and therefore important for improving feed efficiency^37^. In swine, the genus *Succinivibrio* has been considered as a member of the core microbiome of the proximal colon^38^, or cecum^39^, and can metabolize various carbohydrate sources, resulting in fermentation products such as acetate and succinate^40^, forming propionate in further decarboxylation process^41^.

In our study, we obtained heritability estimates for a significant proportion of the investigated OTUs. This corroborates the hypothesis that microbial features are, at least in part, under the direct genetic control of the host. A description of the possible effects of host genetics on gut microbiome has been reported in Maltecca *et al*.^42^. Particularly, the effect of host genetics on OTU determines the heritable portion of gut microbiome. Comparison of OTU heritability estimates across multiple time points show that moderate estimates were obtained at MidTest and OffTest. These findings are consistent with those of Camarinha-Silva *et al*.^43^ who reported similar heritability estimates for genera classified as *Firmicutes* and *Bacteroides* in Piétrain pigs. As pigs age their microbiome composition becomes less dependent on the environment, as reported by Duc *et al*.^19^, which might explain differences in OTU heritability estimates across time points in our study. Interestingly, one taxon classified as *Bacteroides* and two OTUs classified as *Prevotella* were heritable across all three census points, which might be beneficial to the animals because these two groups of bacteria have been proven to be positively associated with the conversion of polysaccharides to short-chain fatty acids^44^, which in turn were found to improve host energy balance, prevent fat deposition in adipose tissues, and promote leptin levels^45^. We also found that the heritable OTUs classified as *Bacteroides* and *Prevotella* were associated with average daily gain and backfat, as previously reported for average daily gain^46^. The two most heritable OTUs in our study were classified as *Peptostreptococcaceae* (h^2^ = 0.55) and *Clostridium butyricum* (h^2^ = 0.46).

Evidence of host-microbiome interaction has been reported in humans, dairy cattle, and mice^47,48^. Furthermore, the possible exploitation of fecal microbial heritability and host genetics in swine selection programs have also been discussed previously^49,50^. To further explore the genomic architecture of host microbiome control, we performed a genome-wide association study. The most significant genomic regions for the gut microbiome were located on SSC11 from 20.7 to 74.5 Mb and on SSC4 from 93.4 to 137.1 Mb. The top SNP *rs80805385* (*P*-value = 7.1 × 10^–24^) and the second most significant SNP *rs341103859* (*P*-value = 5 × 10^−23^) were located on SSC11, while the third most significant SNP *rs81382427 (P*-value = 3 × 10^−21^) was located on SSC4. The genomic regions on SSC4 were significantly associated with 44 markers and OTUs classified as *Lachnospiraceae*. In a previous GWAS on growth and carcass composition on a related population^51^, a significant genomic region associated with average daily gain was identified in the same genomic region.

In this study, within the genomic region on SSC4, we identified the gene *VANGL1*, which is part of the Wnt signaling pathway. This gene is highly expressed in gut tissues and has been linked with cell proliferation/turnover^52^. A study on the gut microbiome of humans reported an association between *VANGL1* and *Sutterellacea* taxa^53^, while in this study was significantly associated with OTU classified as *Anaerostipes*.

Overall, of the 334 OTUs significantly associated with growth and fatness parameters, 18 were heritable and associated with at least one significant marker across time-points (Supplementary Fig. 3a,b). Of these 18 OTUs, 13 were significantly associated with ADG_14-MKT_ at OffTest and classified as *Firmicutes*, *Bacteroidetes*, or *Fusobacteria* (bottom-right panel of Supplementary Fig. 3a).

The gut microbial community of pigs is a rich and complex ecosystem, shaped by a multitude of non-genetic factors, including environment and animal management practices^42^. In this regard, taxonomic abundances are similar to other complex traits in livestock such as fertility and health-related measures. In our study, a sizable proportion of the OTUs investigated showed moderate heritability, suggesting that microbiome composition could be manipulated through selection. Furthermore, we were able to identify several regions of the genome that putatively influence gut microbiome composition in swine. The study presented here is, to the best of our knowledge, one of the first and largest to have investigated the overall impact of the fecal microbiome on growth performance in swine. We did so by exploiting a balanced design as well as a genomically connected population that allowed considerable power in exploring the whole span of variability generated by the host microbial community interaction.

The paucity of similar research in swine makes it difficult to compare the findings of the current study with existing literature. Nonetheless, a recent study of Cheng *et al*.^54^ identified potential genomic regions controlling the abundance of *Ruminococcaceae* on SSC9 and *Turicibacter* on SSC10. Genomic regions affecting a genus within the *Clostridiaceae* family located on SSC11 from 28.2 to 33.5 Mb was found by Crespo-Piazuelo *et al*.^55^, in agreement with the current study. Host genome-microbiome association research performed in humans to date has shown that most loci reported in different studies are not replicated^56^. Genome-wide association studies in various species suggest that host genome-microbiome studies require a large number of variants to be identified reliably^57,58^. In the current work we favored simplicity and easy interpretability by omitting the longitudinal aspect of the experimental design, in effect treating each separate census time as an independent measure. This does not account for the fact that samples are temporally related, and further analyses should try to explicitly model such dependence. Similarly, and for the same reasons, in our analysis we have ignored the compositional nature of microbiome information^19,59^. Since OTU abundance (within time) are not independent from each further research is warranted on how to explicitly model this co-dependence. Since heritability estimates are ratio of variances, changes in heritability over time should be interpreted with caution. As the microbiome matures and pigs transition to a more stable environment moving from the nursery to the growing facility, environmental variability might be reduced, thus increasing the heritability estimates. Specific multiple trait models for the most significant OTUs should be employed to test for a different genetic architecture of microbial composition over time. Finally, as with most analyses, we have only identified associations among the different layers of information available. Further research should elucidate causality among the most significant associations. Recursive modeling as outlined in Maltecca and collegues^42^ and Tsilimigras and collegues^60^ could provide useful information on how to correctly interpret results of associations obtained from multiple sources of information.

This study was performed on a population of crossbred pigs from which fecal samples were collected at three key stages of life. The goal of this study was to estimate heritability of gut bacterial taxa that were important to the host’s growth and fatness, and to identify regions on the host genome that might influence the composition of the gut microbiome. Our results show that host genetic factors play an important role both in determining growth and fatness and in affecting the gut microbiome composition in swine. The significant associations that we identified between gut microbiome features and growth and fatness phenotypes suggest that host genetics may affect these associations. A total of 334 OTUs were significantly associated with growth and fatness parameters across three-time points. The heritability estimates of these OTUs were calculated, and although most were found to be relatively low, a few indicated moderate levels of heritability. Using a genome-wide association study, we discovered host genomic regions and markers associated with taxa that were themselves associated with growth and fatness. The work presented in this paper is one of the first studies of its kind in farm animal research. It is our hope that the findings will inform our understanding of the host genome-microbiome relationship, which in turn might support future research in this field.

## Methods

### Experimental design and sample collection

Phenotypic records presented in this study came from a commercial farm operated by The Maschhoffs LLC (Carlyle, IL, USA). All methods and procedures were in accordance to the Animal Care and Use policies of North Carolina State University and the National Pork Board. The experimental protocol for fecal sample collection received approval number 15027 from Institutional Animal Care and Use Committee. The experimental design was previously reported^19^ and summarized in Supplementary Fig. 4. Briefly, twenty-eight purebred Duroc sires were crossed with Yorkshire × Landrace or Landrace × Yorkshire sows (dam lines) to yield the individuals used in this study. The pigs were weaned at 18.6 ±1.09 days and were moved to a nursery-finishing facility, where they were allotted in groups of 20 individuals per pen. Pen mates were paternal half-siblings of the same gender (gilts or barrows) and of similar weaning weight. The experiment was repeated six times (contemporary groups), each of which comprised one pen of gilts and one pen of barrows from each of the 28 sires. The test period began the day the pigs were moved to the nursery-finishing facility. During the grow-finish period they were fed standard diets based on gender and live weight and received a standard vaccination and medication routine (Supplementary Tables 8 and 9). End of test was reached on a pen-specific basis when all pigs in a pen achieved an average live weight of 138.5 ±12.7 kg, at which point pigs were transported to the abattoir. Rectal swabs were collected from all pigs in a pen at 3 time points: (i) the day after weaning (**Wean**), (ii) 15 weeks post-weaning (**MidTest**; average age 118.2 ±1.18 d), and 22 weeks post-weaning (**OffTest**; average age 174.3 ±1.43 d). The MidTest time point was chosen as representative of the growing phase, when the muscle deposition phases into fat deposition. Four pigs per pen were selected to cover all the within-pen variability in lean carcass growth, and their rectal swabs were used for microbiome sequencing. In the end, the number of samples at Wean, MidTest, and OffTest were 1,205, 1,295, and 1,283, respectively. There were 1,039 animals having samples collected at all 3 time points. More details on the distribution of samples across families, time points, and sex are provided in (Supplementary Table 10). Average daily gain was calculated as difference in live weight from birth to week 14 (**ADG**_**B-14**_), from weaning to week 14 (**ADG**_**W-14**_), from week 14 to week 22 (**ADG**_**14–22**_) and from week 14 to harvest (**ADG**_**14-MKT**_)^20^. Back fat thickness and loin depth were recorded on live animals at weeks 14 and 22 post-weaning, hereafter referred to as **BF**_**14**,_ **BF**_**22**_, **LD**_**14**_, and **LD**_**22**_, respectively. There were 1,028 animals with microbiome data at Wean, MidTest, and OffTest, as well as records of ADG_B-14_, ADG_W-14_, ADG_14–22_, ADG_14-MKT_, BF_14_, BF_22_, LD_14_, and LD_22_. These were the animals used for all subsequent analyses in this study. Descriptive statistics of the phenotypic records are presented in Table 1. All individuals in the study were genotyped with the Illumina PorcineSNP60 BeadChip (Illumina Inc., San Diego, CA USA). All samples had call rates of at least 0.90. A SNP minor allele frequency filter of 0.02 was applied. A total of 40,542 SNPs distributed over all 18 autosomes remained for subsequent analyses. After QC steps 1,028 animals with both phenotypes genotypes and microbiome data were available.

**Table 1:** Mean and standard deviation (SD) of phenotypic measurements from crossbred pigs (n = 1,039).

Traits	Mean	SD
*Growth:*		
ADG_B-14_, kg/d	0.57	0.08
ADG_W-14_, kg/d	0.64	0.10
ADG_14-22_, kg/d	0.87	0.16
ADG_14-MKT_, kg/d	0.89	0.14
*Fatness:*		
BF_14_, mm	12.5	2.81
BF_22_, mm	20.1	5.36
LD_14_, mm	42.3	4.80
LD_22_, mm	55.8	5.15

ADG_B-14_ = average daily gain from birth to week 14 post-weaning; ADG_W-14_ = average daily gain from weaning to week 14 post-weaning; AD_G14–22_ = average daily gain from weeks 14 to 22 post-weaning; AD_G14-MKT_ = average daily gain from week 14 post-weaning to market; BF = back fat thickness measured at 14 or 22 weeks post-weaning; LD = loin depth measured at 14 or 22 weeks post-weaning.

### DNA extraction sequencing, sequencing, OTU picking, and quality control of data

Total genomic DNA (gDNA) was extracted as described in detail by^19^ and Additional file 1. All sequencing was performed at the DNA Sequencing Innovation Lab at the Center for Genome Sciences and Systems Biology at Washington University in St. Louis (USA). As previously described by^19^, raw data was transformed into OTU counts through two sets of steps. In the first set of steps, pairs of V4 16 S rRNA gene sequences were merged using FLASh (v1.2.11; min overlap = 100; max overlap = 250), after which merged sequences were subjected to quality-based filtering using PRINSEQ (v0.20.4; min mean qual = 35). Merged sequences that survived quality filtering were oriented in a common direction, and primer sequences were identified and trimmed. Sequences were demultiplexed using the split_libraries_fastq.py script (QIIME v1.9.1). In the second set of steps, sequences with >97% nucleotide sequence identity were clustered into operational taxonomic units (“OTUs”) using pick_open_reference_otus.py (QIIME v1.9.1;–suppress_step4; -s 0.1; align_seqs:min_length 75; pick_otus:similarity 0.97; pick_otus:max_accepts 50; pick_otus:max_rejects 8). A modified version of GreenGenes (May 2013) was used as the reference database. To generate this modified database, sequences from the original GreenGenes reference were trimmed to the V4 region, dereplicated, filtered for sequences containing ambiguous bases, sorted by descending length, and clustered de novo at >97% sequence identity using UCLUST (v1.2.21q) with default settings. The most abundant sequence in each cluster was selected as the representative sequence for that cluster. 10% of the reads with no hit to this reference database were then clustered de novo with UCLUST to generate new reference OTUs to which the remaining 90% of reads were assigned. The most abundant sequence in each cluster was used as the representative sequence for the OTU. Sparse OTUs were filtered out by requiring a minimum total observation count of 1,200 for an OTU to be retained, and the OTU table was rarefied to 10,000 counts per sample. Finally, the Ribosomal Database Project (RDP) classifier (v2.4) was retrained in the manner described in^45^, and a bootstrap cutoff value of 0.8 was used to assign taxonomy to the representative sequences. The final data set was composed of 1,678 OTUs. Average Good’s coverage estimates for samples at Wean, MidTest and OffTest were 0.99 ± 0.002, 0.98 ± 0.002, and 0.98 ± 0.002, respectively. The R package “vegan”^61^ was used to measure alpha diversity in this study. Alpha diversity was calculated using the Shannon diversity index as $-{\Sigma }_{i=1}^{n}{p}_{i}\,\mathrm{ln}({p}_{i})$, where *p*_*i*_ was the proportional abundance of OTU_i_.

### Data analysis

Analysis of the data was carried out in three parts. In the first part, OTU abundances obtained at Wean, MidTest, and OffTest (1,678 OTU at each time point) were evaluated for their associations with the following traits: ADG_B-14_, ADG_W-14_, ADG_14–22_, ADG_14-MKT_, BF_14_, BF_22_, LD_14_, and LD_22_. In the second part of the analysis, heritability estimates were obtained for all OTUs at the three census points. Lastly, a genome-wide association study was performed for those OTU/time combinations for which heritability estimates were higher than an arbitrary threshold of 0.075 and for which the 95% confidence interval did not include zero. An overall representation of the experimental design is depicted in Supplementary Fig. 4.

### OTU-phenotype associations

OTU counts were fitted as covariates in the following model to identify those that had a significant association with traits of interest at a particular time point:

1 $${\bf{y}}={\bf{Xb}}+{{\boldsymbol{\gamma }}}_{{\bf{f}}}{\bf{OT}}{{\bf{U}}}_{{\bf{f}}}+{\bf{Zp}}+{\bf{e}}$$

where **y** was a vector of phenotypic values; **b** was a vector of fixed effects including sex (n = 2), sire (n = 28), dam line (n = 2), and contemporary group (n = 6); ${{\boldsymbol{\gamma }}}_{{\bf{f}}}$ was the regression coefficient for the f^th^ OTU; **OTU**_**f**_ was the count for the f^th^ OTU; **p** was a vector of random pen effects; and **e** was a vector of random residuals. Effects **p** and **e** were assumed to be uncorrelated and had zero mean, and variances ${{\rm{\sigma }}}_{{\rm{p}}}^{2}$ and ${{\rm{\sigma }}}_{{\rm{e}}}^{2}$, respectively. **X** and **Z** were the corresponding incidence matrices. Each association analysis was performed using the “lme4”^62^ package in R (http://www.R-project.org/). A false discovery rate^63^ of 5% (**FDR**) was used to declare a significant association with the trait. The proportion of phenotypic variance absorbed by each OTU regression was calculated as $[{\rm{var}}({{\rm{\gamma }}}_{{\rm{f}}}{{\rm{OTU}}}_{{\rm{f}}})/{\rm{var}}({\rm{y}})]$.

### Heritability estimates

A sire model was fitted to estimate variance components and heritability estimates for 1,678 OTU at Wean, MidTest, and OffTest. The model used was:

2 $${\bf{y}}={\bf{Xb}}+{\bf{Zs}}+{\bf{Wp}}+{\bf{e}}$$

where **y** is a vector of OTU counts (n = 1,678); **b** was a vector of the fixed effects highlighted above; **s** was the vector of random effects of sire, and **p** and **e** were as in the previous model. The random effects, **s**, **p**, and **e** were assumed to be uncorrelated to one another, and to have zero mean, and variance of ${{\rm{\sigma }}}_{{\rm{s}}}^{2}$, ${{\rm{\sigma }}}_{{\rm{p}}}^{2}$, and ${{\rm{\sigma }}}_{{\rm{e}}}^{2}$, respectively. Sires were in this case assumed uncorrelated based on both their pedigree as well as genomic relationships (results not shown). **X**, **Z**, and **W** were the corresponding incidence matrices. Heritability estimates and the standard error of the estimate for each taxon were computed according to^64^ as:

$${{\rm{h}}}^{2}=\frac{4{{\rm{\sigma }}}_{{\rm{s}}}^{2}}{({{\rm{\sigma }}}_{{\rm{p}}\,}^{2}+{{\rm{\sigma }}}_{{\rm{s}}\,}^{2}+{{\rm{\sigma }}}_{{\rm{e}}\,}^{2})}$$

$${\rm{SE}}=\frac{16{{\rm{\sigma }}}_{{\rm{s}}}^{2}}{{({{\rm{\sigma }}}_{{\rm{p}}}^{2}+{{\rm{\sigma }}}_{{\rm{s}}}^{2}+{{\rm{\sigma }}}_{{\rm{e}}}^{2})}^{2}}$$

### Alpha diversity and OTU genome-wide association

A genome-wide association analysis was performed to identify markers significantly associated with either alpha diversity or individual taxa at each of the three census points. The model used was:

3 $${\bf{y}}={\bf{Xb}}+{{\boldsymbol{\gamma }}}_{{\bf{f}}}{\bf{SN}}{{\bf{P}}}_{{\bf{f}}}+{\bf{Za}}+{\bf{Wp}}+{\bf{e}}$$

where **y** was a vector of alpha diversity or OTU; **b** was as before; ${{\boldsymbol{\gamma }}}_{{\bf{f}}}$ was the regression coefficient on the number of copies of the minor allele for the f^th^ SNP (from 1 to 40,542 coded as 0, 1, 2); **a** was the animal polygenic effect distributed as N(0, ${\bf{G}}{{\rm{\sigma }}}_{{\rm{a}}}^{2}$), with **G** being the genomic relationship matrix was constructed following the method reported by VanRaden, 2008^65^ and ${{\rm{\sigma }}}_{{\rm{a}}}^{2}$ the additive genomic variance; **p** and **e** were as before. **X, Z** and **W** were the corresponding incidence matrices. An FDR of 5% was once again employed. Significant SNPs were above the threshold of *P*-value <1 × 10^−5^. The analysis was performed using “rrblup” package^66^ of the R software. Based on the position of significant markers, previously discovered quantitative traits loci (**QTL**) were co-localized using the *Sus scrofa* 11.1 assembly and the AnimalQTLdb^67^ database. Gene annotations were obtained using the position of each significant marker and the Biomart platform on Ensembl^68^ through the ‘biomaRt’ R package^69^. A gene ontology analysis was carried out using Database for Annotation, Visualization and Integrated Discovery (DAVID) Bioinformatics Resources version 6.7^70^.

## Supplementary information

Supplementary information

Supplementary Figure S1

Supplementary Figure S2

Supplementary Figure S3

Supplementary Figure S4

Supplementary Table S1

Supplementary Table S2

Supplementary Table S3

Supplementary Table S4

Supplementary Table S5

Supplementary Table S6

Supplementary Table S7

Supplementary Table S8

Supplementary Table S9

Supplementary Table S10

## Data Availability

The microbiome data that support the findings in this study are available from MATATU but restrictions apply to the availability of these data, which were used under license for the current study, and so are not publicly available. Data are, however, available from the authors upon reasonable request and with permission of MATATU.
