# Supplementary material for: Heritability and genome-wide association of swine gut microbiome features with growth and fatness parameters
Source: Sci Rep. 2020 Jun 23;10:10134. doi: 10.1038/s41598-020-66791-3 (PMC7311463; doi:10.1038/s41598-020-66791-3)
Supplement: Supplementary file 1 — Supplementary information [file 41598_2020_66791_MOESM1_ESM.docx]

**Additional file 1 for manuscript:**

Heritability and genome-wide association of swine gut microbiome features with growth and fatness parameters

Matteo Bergamaschi^1^, Christian Maltecca^1^, Constantino Schillebeeckx^2^, Nathan P. McNulty^2^, Clint Schwab^3^, Caleb Shull^3^, Justin Fix^3^, Francesco Tiezzi^1*^

^1^Department of Animal Science, North Carolina State University, Raleigh, NC 27695, USA.

^2^Matatu, Inc., 4340 Duncan Ave., Suite 211, St. Louis, MO 63110, USA.

^3^The Maschhoffs LLC, Carlyle, IL 62231, USA.

**DNA extraction and purification**

Total genomic DNA (gDNA) was extracted as described in detail by ^19^. Briefly, 650 µL of extraction buffer (200 mM Tris; 200 mM NaCl; 20 mM EDTA, pH 8.0) was added to each swab stored in a 2 mL self-standing screw cap tube (Axygen, CA, USA). Tubes were shaken using a Mini-BeadBeater-96 (MBB-96; BioSpec, OK, USA) for 20 s to free sample material from the swab head. Following a brief centrifugation (10 s; 500 × *g*) to pull down any dislodged material, each swab head was removed from its tube using sterile forceps. Samples were frozen solid at –80 °C, and approximately 250 µL of 0.1 mm zirconia/silica beads (BioSpec) and a 3.97 mm stainless steel ball were added to the sample (while still frozen, to avoid splashing). Samples were allowed to thaw briefly, after which 210 µL 20% SDS and 500 µL phenol:chloroform:IAA (25:24:1, pH 8.0) were added. Bead-beating was performed on the MBB-96 (4 min; room temperature), samples were centrifuged (3,220 × *g*; 4 min), and 250 µL of the aqueous phase was transferred to a new tube. 100 µL of this crude DNA was then further purified using a QIAquick 96 PCR purification kit (Qiagen, MD, USA). Purification was performed per the manufacturer’s instructions with the following minor modifications: (i) sodium acetate (3 M, pH 5.5) was added to Buffer PM to a final concentration of 185 mM to ensure optimal binding of genomic DNA to the silica membrane; (ii) crude DNA was combined with 4 volumes of Buffer PM (rather than 3 volumes); and, (iii) DNA was eluted in 100 µL Buffer EB (rather than 80 µL).

**Illumina library preparation and sequencing**

Phased, bi-directional amplification of the V4 region (515–806) of the 16S rRNA gene was employed to generate indexed libraries for Illumina sequencing using the strategy described in ^63^. Amplicon libraries were quantified using the Qubit dsDNA assay kit (Thermo Fisher Scientific Inc., MA, USA) before being pooled in equimolar ratios. These final pools were purified using Agencourt AMPure XP beads (Beckman Coulter) per the manufacturer’s instructions. Purified pools were supplemented with 5-10% PhiX control DNA and were sequenced on an Illumina MiSeq machine as paired-end 2×250 + 13bp index reactions using the 600v3 kit. Un-demultiplexed FASTQ files were generated by MiSeq Reporter. All sequencing was performed at the DNA Sequencing Innovation Lab at the Center for Genome Sciences and Systems Biology at Washington University in St. Louis (USA).

**16S rRNA gene sequencing and quality control of data**

Pairs of V4 16S rRNA gene sequences were first merged into a single sequence using FLASh v1.2.11^58^, with a required overlap of at least 100 and not more than 250 base pairs in order to provide a confident overlap. Sequences with a mean quality score below Q35 were then filtered out using PRINSEQ v0.20.4^58^. Sequences were oriented in the forward direction and any primer sequences were matched and trimmed off; during primer matching, up to 1 mismatch was allowed. Sequences were subsequently de-multiplexed using QIIME v1.9^59^. Sequences with >97% nucleotide sequence identity were then clustered into operational taxonomic units (hereafter “**OTU**”) using QIIME with the following settings: *max_accepts = 50, max_rejects = 8, percent_subsample = 0.1 and --suppress_step4*. A modified version of GreenGenes^60,61^ was used as the reference database. Input sequences that had 10% of the reads with no hit to the reference database were then clustered de novo with UCLUST^64^ to generate new reference OTU to which the remaining 90% of reads were assigned. The most abundant sequence in each cluster was used as the representative sequence for the OTU. Sparse OTU were then filtered out by requiring a minimum total observation count of 1,200 for an OTU to be retained, and the OTU table was rarefied to 10,000 counts per sample. The final data set was composed of 1,678 OTUs. Average Good’s coverage estimates for samples at Wean, MidTest and OffTest were 0.99 ±0.002, 0.98 ±0.002, and 0.98 ±0.002, respectively. Finally, the Ribosomal Database Project (RDP) classifier (v2.4) was retrained in the manner described in ^44^, and a bootstrap cutoff value of 0.8 was used to assign taxonomy to the representative sequences. The R package “vegan”^62^ was used to measure alpha diversity in this study. Alpha diversity was calculated using the Shannon diversity index as $-\Sigma_{i=1}^{n}p_{i}ln(p_{i})$, where *p_i_* was the proportional abundance of OTU_i_.

**Reference:**

19. Lu, D. *et al.* Host contributes to longitudinal diversity of fecal microbiota in swine selected for lean growth. *Microbiome* **6**, (2018).

44. Ridaura, V. K. *et al.* Gut Microbiota from Twins Discordant for Obesity Modulate Metabolism in Mice. *Science* **341**, 1241214–1241214 (2013).

58. Schmieder, R. & Edwards, R. Quality control and preprocessing of metagenomic datasets. Bioinformatics 27, 863–864 (2011).

59. Caporaso, J. G. et al. QIIME allows analysis of high-throughput community sequencing data. Nature Methods 7, 335–336 (2010).

60. Schloss, P. D. & Handelsman, J. Toward a Census of Bacteria in Soil. PLoS Computational Biology 2, e92 (2006).

61. Ley, R. E., Turnbaugh, P. J., Klein, S. & Gordon, J. I. Human gut microbes associated with obesity. Nature 444, 1022–1023 (2006).

62. Oksanen, J. *et al.* vegan: Community Ecology Package. R package version 2.5-6. (2019).

63. Faith, J. J. *et al.* The Long-Term Stability of the Human Gut Microbiota. *Science* **341**, 1237439 (2013).

64. Edgar, R. C. Search and clustering orders of magnitude faster than BLAST. *Bioinformatics* **26**, 2460–2461 (2010).
