# Supplementary material for: Heritability and genome-wide association of swine gut microbiome features with growth and fatness parameters
Source: Sci Rep. 2020 Jun 23;10:10134. doi: 10.1038/s41598-020-66791-3 (PMC7311463; doi:10.1038/s41598-020-66791-3)

Supplementary Figure 1.

Proportional representation of bacterial families in the fecal microbiome at Wean, MidTest, and OffTest.

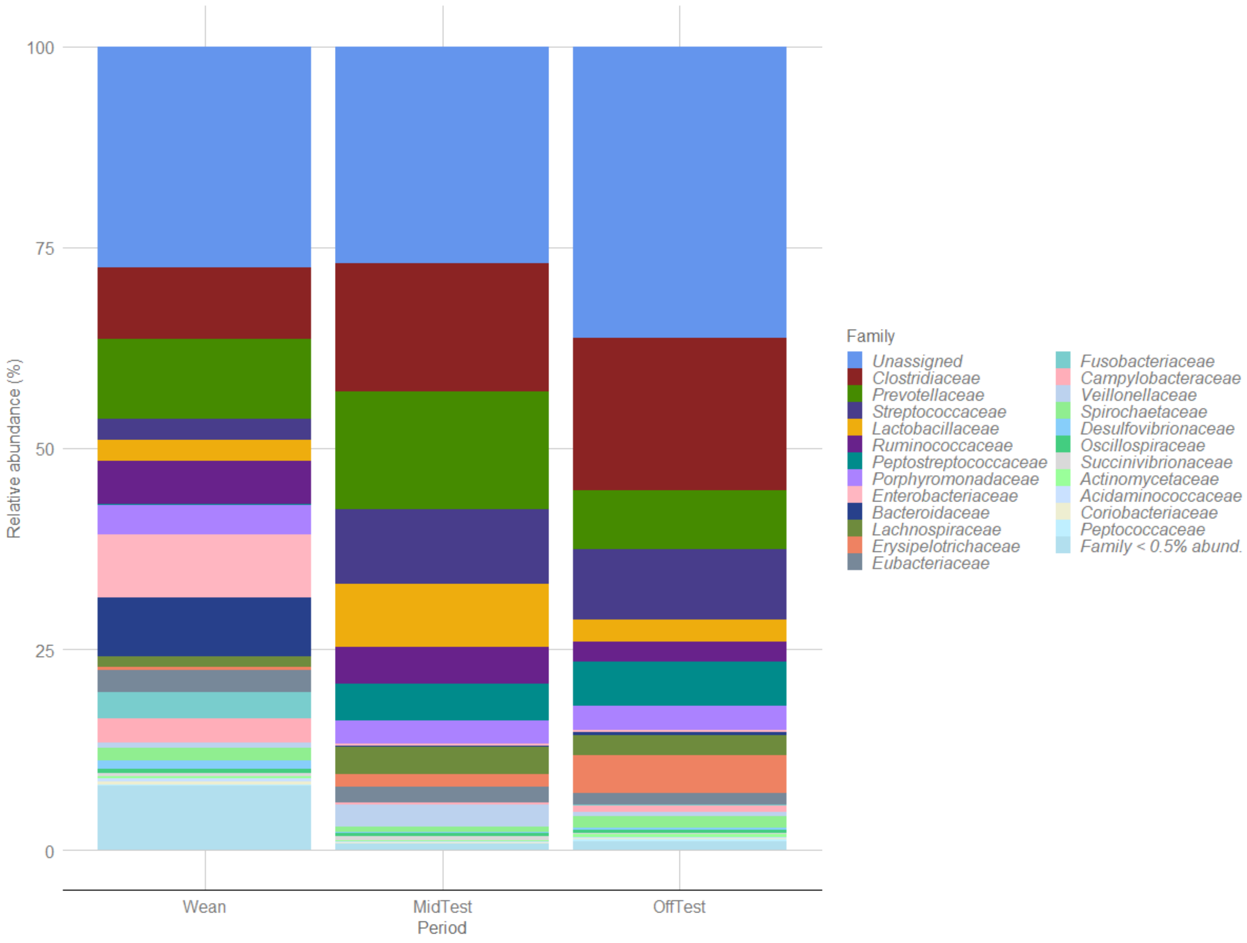

Supplement: Supplementary file 2 — Supplementary Figure S1 [file 41598_2020_66791_MOESM2_ESM.pdf]
