# Supplementary material for: Heritability and genome-wide association of swine gut microbiome features with growth and fatness parameters
Source: Sci Rep. 2020 Jun 23;10:10134. doi: 10.1038/s41598-020-66791-3 (PMC7311463; doi:10.1038/s41598-020-66791-3)
Supplement: Supplementary file 3 — Supplementary Figure S2 [file 41598_2020_66791_MOESM3_ESM.pdf]

Supplementary Figure 2.

Manhattan plots for genome wide association analysis of alpha diversity at Weaning, MidTest and OffTest. The horizontal line indicates the threshold for genome-wide significance ($P < 1\times10^{-5}$).

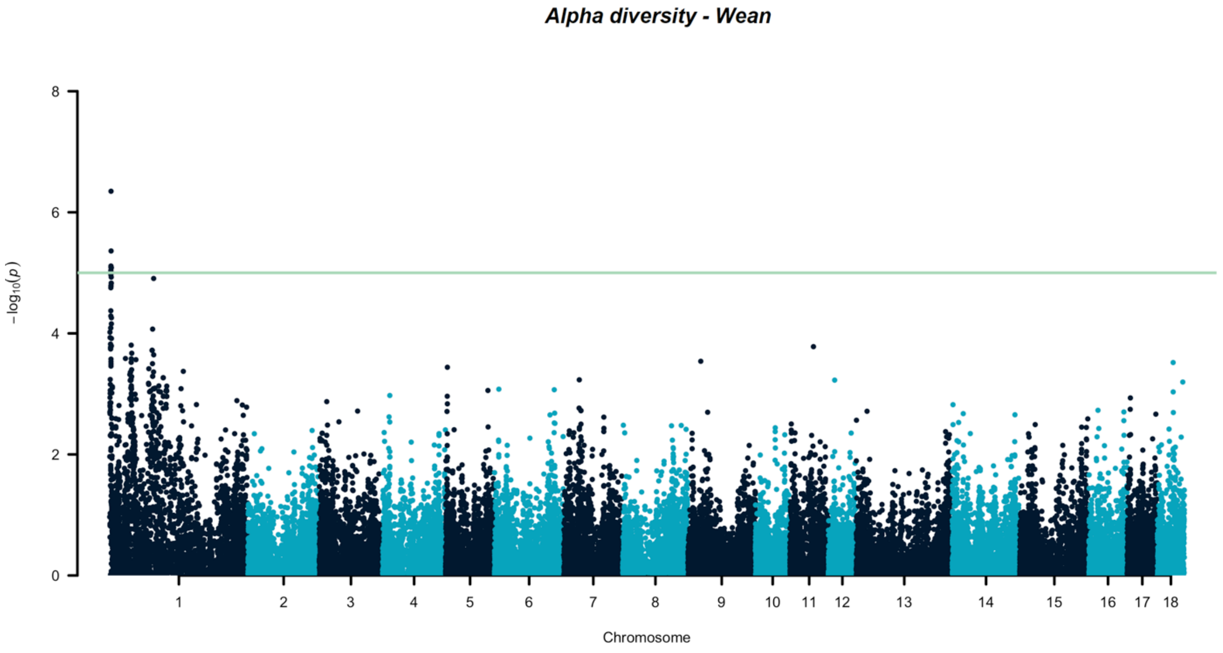

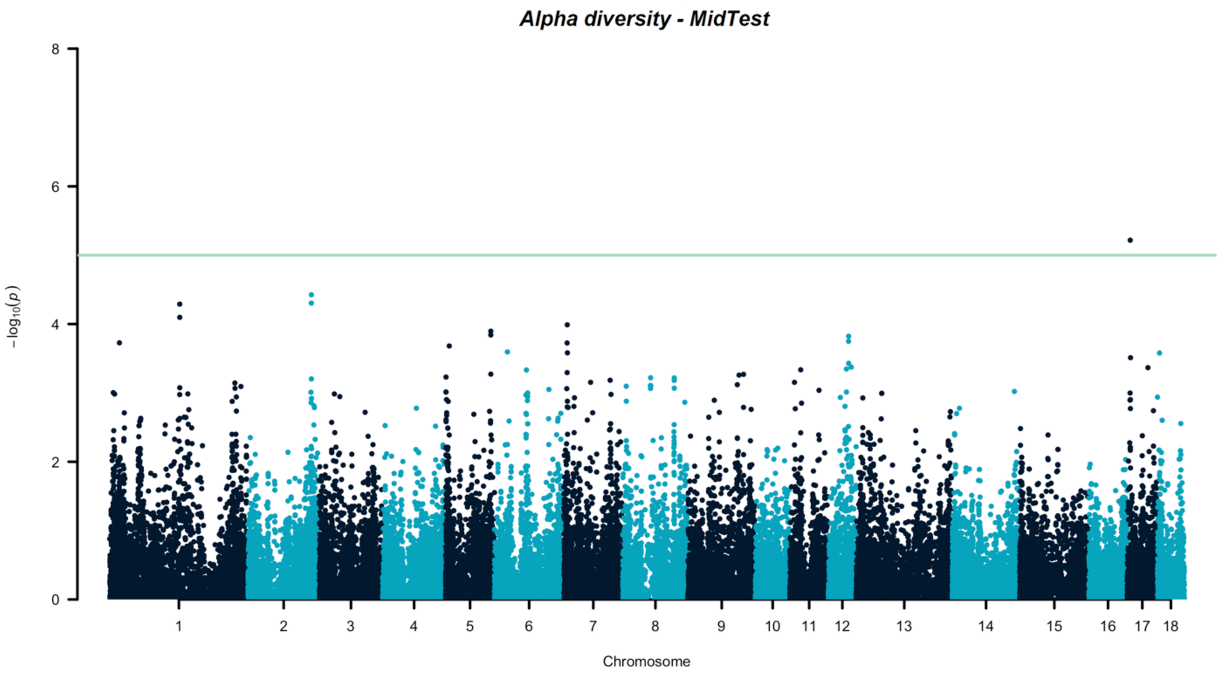
Alpha diversity - MidTest
8
6
4
2
0
$-\log_{10}(p)$
1
2
3
4
5
6
7
8
9
10
11
12
13
14
15
16
17
18
Chromosome

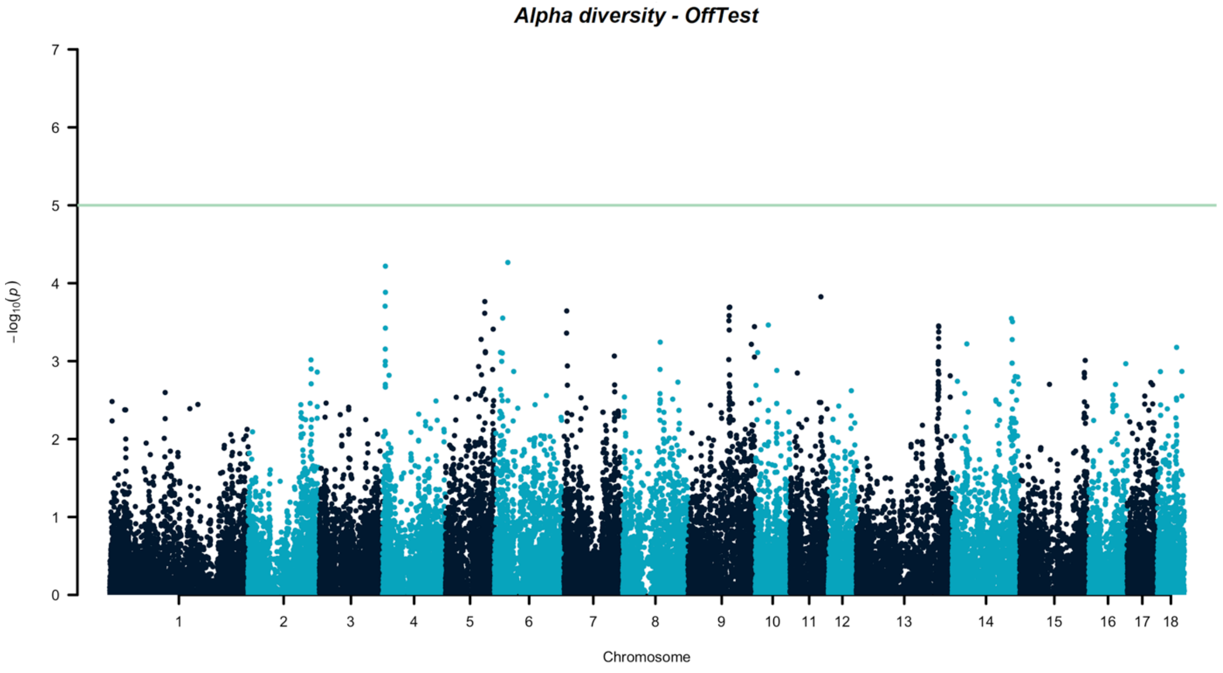
Alpha diversity - OffTest
7
6
5
4
3
2
1
0
$-\log_{10}(p)$
1
2
3
4
5
6
7
8
9
10
11
12
13
14
15
16
17
18
Chromosome
