# Supplementary material for: Heritability and genome-wide association of swine gut microbiome features with growth and fatness parameters
Source: Sci Rep. 2020 Jun 23;10:10134. doi: 10.1038/s41598-020-66791-3 (PMC7311463; doi:10.1038/s41598-020-66791-3)
Supplement: Supplementary file 4 — Supplementary Figure S3 [file 41598_2020_66791_MOESM4_ESM.pdf]

Supplementary Figure 3.

Distribution of abundance of taxa significantly associated (As) with growth and fatness parameters, hereditable (Her), and with at least one significant marker at Wean (top), MidTest (middle), and OffTest (bottom).

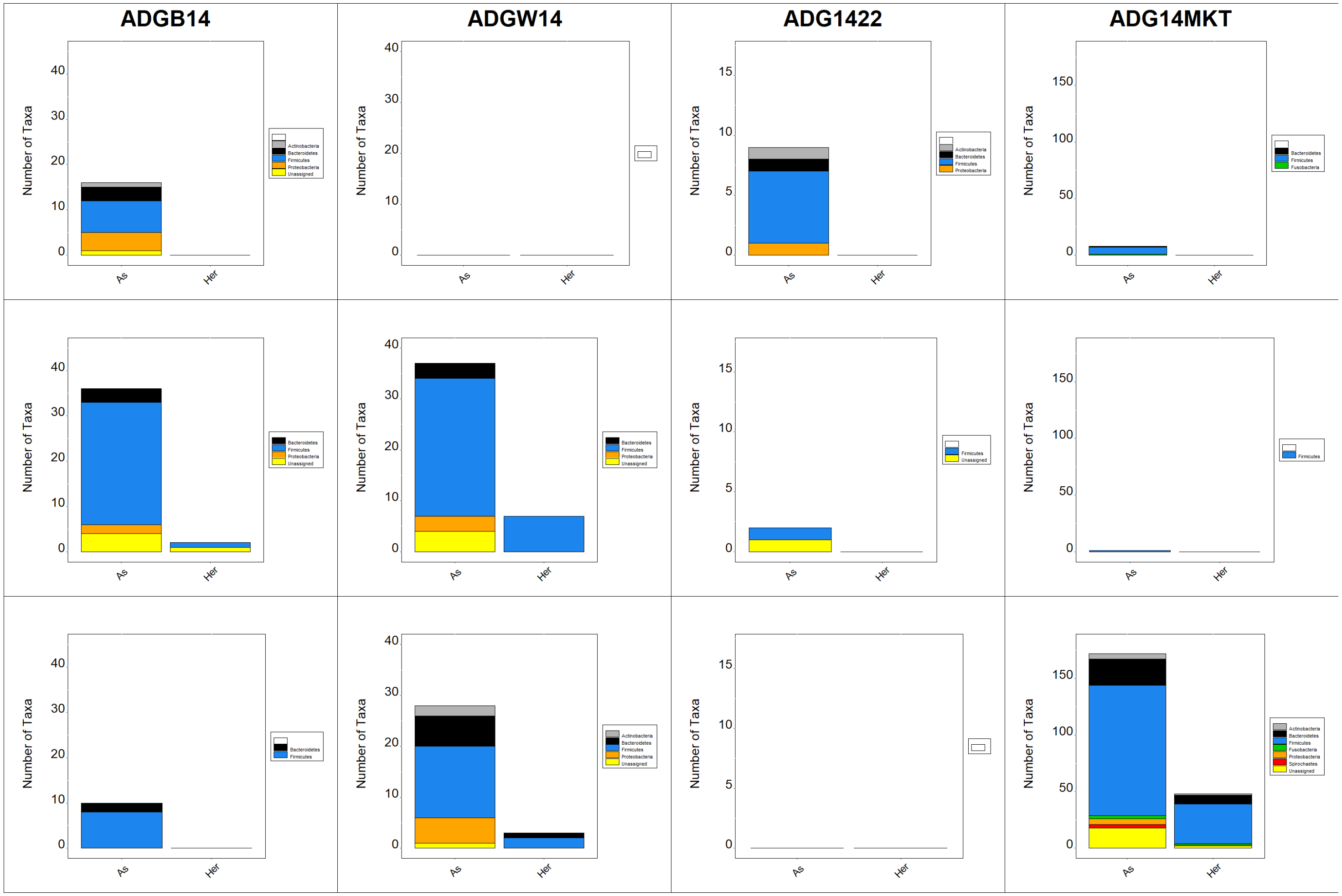

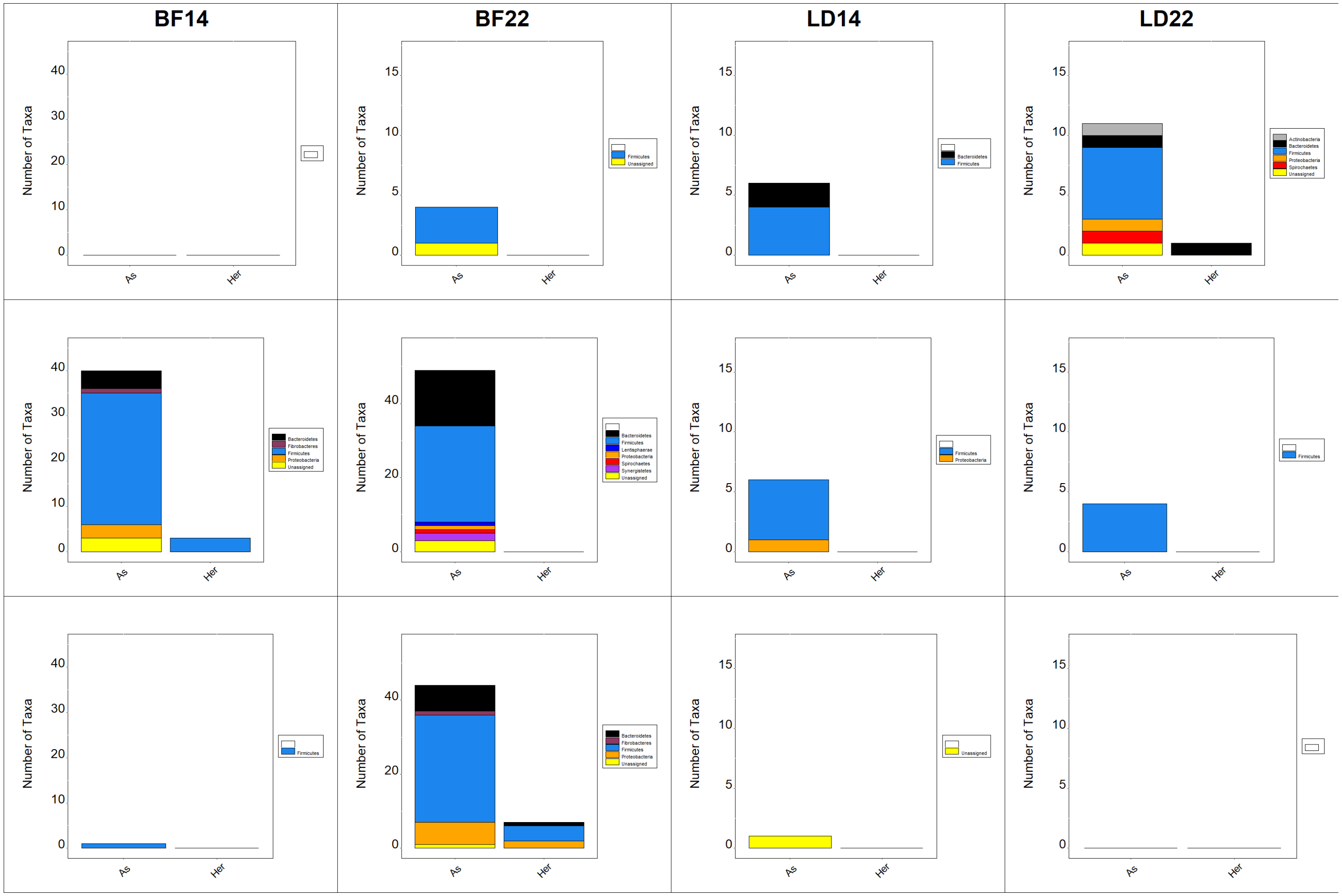
BF14
BF22
LD14
LD22
Number of Taxa
As
Her
Bacteroidetes
Fibrobacteres
Firmicutes
Proteobacteria
Unassigned
Lentisphaerae
Spirochaetes
Synergistetes
Actinobacteria
