# Supplementary figures and images for: Heritability and genome-wide association of swine gut microbiome features with growth and fatness parameters
Source: Sci Rep. 2020 Jun 23;10:10134. doi: 10.1038/s41598-020-66791-3 (PMC7311463; doi:10.1038/s41598-020-66791-3)

# Supplementary Figure 4.

Flow chart of the analytical overview of this study.

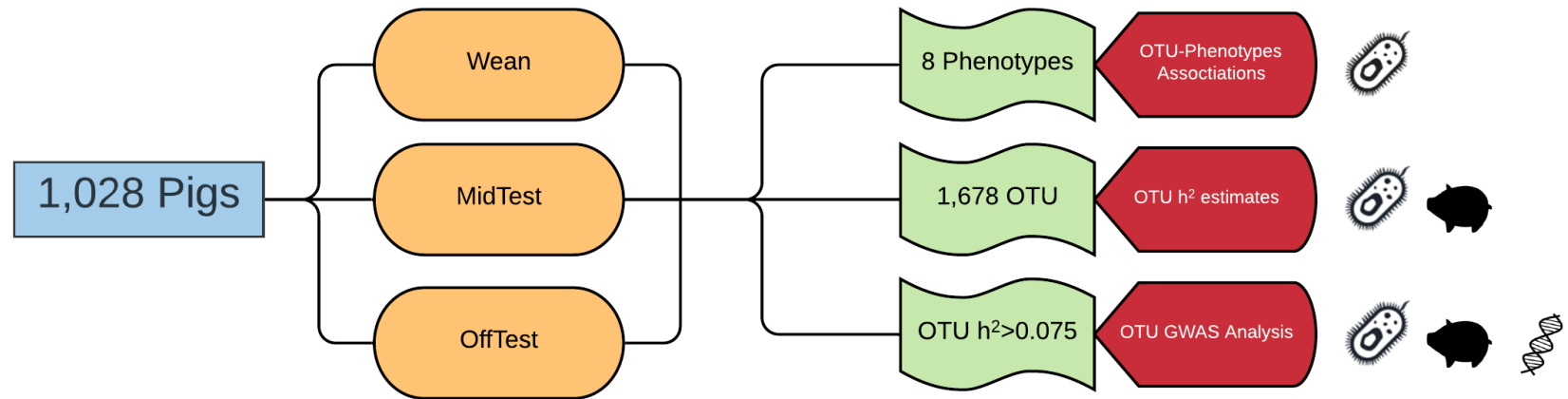

Supplement: Supplementary file 5 — Supplementary Figure S4 [file 41598_2020_66791_MOESM5_ESM.pdf]
